# Drought resistance mechanisms of *Phedimus aizoon* L.

**DOI:** 10.1038/s41598-021-93118-7

**Published:** 2021-06-30

**Authors:** Yuhang Liu, Zhongqun He, Yongdong Xie, Lihong Su, Ruijie Zhang, Haixia Wang, Chunyan Li, Shengju Long

**Affiliations:** 1 College of Horticulture, Sichuan Agricultural University, Chengdu, 611130 People’s Republic of China; 2 Institute for Processing and Storage of Agricultural Products, Chengdu Academy of Agricultural and Forest Sciences, Chengdu, 611130 People’s Republic of China

**Keywords:** Photosynthesis, Plant physiology

## Abstract

*Phedimus aizoon* L. is a drought-resistant Chinese herbal medicine and vegetable. However, its drought tolerant limit and the mechanism of drought tolerance are unknown, which restricts the promotion of water-saving cultivation of *Phedimus aizoon* L. in arid areas. To solve the above problem, we carried out a 30-day-long drought stress experiment in pots that presented different soil water contents and were divided into four groups: control check, 75–80% of the maximum water-holding capacity (MWHC); mild drought, 55–60%; moderate drought, 40–45%; and severe drought, 20–25%. The dynamic changes in both plant physiological indexes from 10 to 30 days and leaf anatomical structure on the 30th day of stress were recorded. The results show that *Phedimus aizoon* L. grew normally under mild drought stress for 30 days, but the growth of the plants became inhibited after 20 days of severe drought and after 30 days of moderate drought. At the same time, *Phedimus aizoon* L. physiologically responded to cope with drought stress: the growth of the root system accelerated, the waxy layer of the leaves thickened, and the dark reactions of the plants transformed from those of the C3 cycle to CAM. The activity of antioxidant enzymes (SOD, POD and CAT) continuously increased to alleviate the damage caused by drought stress. To ensure the relative stability of the osmotic potential, the contents of osmoregulatory substances such as proline, soluble sugars, soluble protein and trehalose increased correspondingly. Although *Phedimus aizoon* L. has strong drought stress resistance, our experimental results show that the soil available water content should not be less than 27% during cultivation.

## Introduction

As a worldwide problem, drought is one of the most important stress factors limiting plant growth and yields in arid and semiarid regions^1, 2^. Today, global arid regions account for one-third of the total land area. Drought is considered a multidimensional stress that restricts plant growth and development and causes a series of changes in the physiological, morphological, biochemical and molecular characteristics of plants^3, 4^. The effects of drought stress predominantly vary with plant species and genotype, developmental stage, duration, and stress severity^5, 6^. Generally, although root growth proliferates, plant responses to drought stress are mostly reflected in loss of turgor; drooping, wilting and yellowing of the leaves; decreased plant height, shoot dry weight, and leaf area index; and even damage to blade structure^7–9^. In addition, drought stress causes oxidative stress in plants through the production of reactive oxygen species (ROS), which leads to metabolic disorders and disruption of ion absorption and translocation^10, 11^.

Studies have shown that plant drought stress resistance has a certain correlation with anatomical structure^12^. In addition, the drought stress resistance of plants is mainly manifested by a combination of osmoregulatory substances, changes in membrane lipid components, free radical scavenging, and protein-induced hormone regulation^13^. Drought stress causes an imbalance between the generation and scavenging of active oxygen in plants, while the accumulation of biological free radicals causes membrane damage and membrane lipid peroxidation. Antioxidant defense systems consisting of a series of antioxidant enzymes and osmotic regulators (such as soluble sugars, soluble protein, free proline and trehalose in plants) not only remove these reactive oxygen species but also help maintain turgor and protect the macromolecular structure of cells^14, 15^.

*Phedimus aizoon* L. is a perennial herbaceous species of Crassulaceae family and is widely planted in northern and southern China. It is used for road and roof greening because of its dwarf growing habit, long flowering and vegetative periods, and strong resistance to stress and infertility^16^. In addition to its strong ornamental value, it also has good edible and medicinal value. *Phedimus aizoon* L. is a wild vegetable species selected via traditional breeding and is rich in triterpenes, sterols, flavonoids and other active substances^17^. It is used in foods primarily for its health benefits, such as protecting cardiovascular and cerebrovascular function, promoting blood circulation and enhancing immunity^18^, and this species has become a healthy vegetable with high economic value. *Phedimus aizoon* L. can reproduce asexually quickly and efficiently, planting it in arid areas can help prevention of soil erosion, moreover, the plants themselves can not only be used as green decoration, but also as herbs with medicinal value and vegetables with health care function. In arid areas of China where pasture is scarce, we are trying to add it to cow fodder. To popularize and cultivate it in arid areas, we need to quantify its resistance ability to drought with soil available water content in which it can grow normally, so that we can determine in which arid areas in China are suitable for its growth, how much water it needs to grow properly, to guide water-saving cultivation. At the same time, we want to reveal the physiological mechanism of its drought resistance, so as to provide some technical means for its cultivation in arid areas, in order to obtain higher yield and quality.

## Materials and methods

### Materials

*Phedimus aizoon* L. plants were obtained from cuttings and grown for 20 days in mid-May. These plants concurrently grow rapidly just before flowering (for approximately 60 days before flowering). The seedlings were provided by the College of Horticulture, Sichuan Agricultural University (30° 42′ N, 103° 51′ E).

### Experimental design and moisture control

The experiment was conducted from May 2019 to October 2019 at the Chengdu Campus of Sichuan Agricultural University. Before the experiment, nutrient-enriched soil consisting of humus soil:perlite:vermiculite = 4:1:1 (V:V:V) was dried to constant weight, and then plastic pots (14 cm in diameter and height) were filled with the nutrient-enriched soil. The quality of dried soil and soil saturated with water was recorded, which was repeated five times, and the maximum water-holding capacity (MWHC) was calculated. Seedlings of *Phedimus aizoon* L. exhibiting the same growth were then selected and transplanted into pots filled with the same quantity of dried nutrient-enriched soil.

Drought stress was simulated by the soil-weighing method. Four drought stress levels were applied in the test: nonstress (75–80% of the MWHC, control), mild drought stress (55–60% of the MWHC, T1), moderate drought stress (40–45% of the MWHC, T2) and severe drought stress (20–25% of the MWHC, T3). The water was replenished every morning and evening, the amount of which was determined by weighing the soil, and a soil three-parameter instrument (WET-2, Zealquest Scientific Technology Co., Ltd., China) was used to monitor soil moisture. The parameters of the plants and soil used in the experiment are listed in Tables 1 and 2.

**Table 1:** Test soil moisture content information.

Moisture control	MWHC	W_hc_ (%)	W_hc_ (mm)	AWC (mm)
CK	75–80%	61.00	81.98	73.92
T1	55–60%	46.00	61.82	53.76
T2	40–45%	33.00	44.35	36.29
T3	20–25%	18.00	24.19	16.13

The average soil water content was obtained from several random samples (n = 5).

MWHC, maximum water-holding capacity; W_hc_, soil water-holding capacity; AWC, available water content = (W_hc_ − W_pwp_) * (ρb/ρw) * Hr.

**Table 2:** Test soil and plant information.

ρb (g/cm^3^)	W_pwp_ (%)	W_pwp_ (mm)	Hs (mm)	Hr (mm)	ρw (g/cm^3^)
1.12	6	8.06	120	120	0.998

ρb, soil bulk density; W_pwp_, permanent wilting point; Hs, average soil depth; Hr, root length; Ρw, water density (at 20 °C).

Each treatment involved three replicates, and each replicate comprised 10 plants, which were randomly plant in pots and placed 10 cm apart. The pots were kept under ambient environmental conditions with natural sunlight and temperature in a plastic house. Air temperature ranged from 28 ± 2.5 °C (day) to 20 ± 2.5 °C (night). Ten plants of each treatment were randomly selected at 10 d, 20 d, and 30 d at 9:00 in the morning (3rd to 5th leaves from top to bottom) for determination of various physiological indicators. Three plants were randomly selected on 30th day at 9:00 a.m. to observe the anatomical structure of the leaves.

### Experiment methods

In this study, all the methods we used were performed in accordance with the relevant guidelines and regulations.

#### Determination of growth parameters

Whole plants were gently uprooted and divided into shoots and roots, rinsed with tap water and washed repeatedly again with deionized water. Plant height and root length were measured with a millimeter-scale ruler, and stem diameter was measured with Vernier calipers.

#### Determination of the relative water content of the leaves

The RWC was calculated using the formula RWC = [(Fresh mass − dry mass)/(Turgid mass − dry mass)] × 100. Each sample comprised all the leaves of a plant.

#### Determination of lipid peroxidation and osmolyte levels

Malondialdehyde (MDA) contents were measured to determine the level of lipid peroxidation according to the method used by Kumar and Knowles^19^. Electrolyte leakage was measured following the method used by Dionisio-Sese and Tobita^20^. The soluble sugar content was measured by the anthranone-ethyl acetate method^21^, and the proline content was assayed by the sulfo-salicylic acid method^22^. The soluble protein content was measured by the Coomassie brilliant blue method^23^.

The accumulation of trehalose was determined according to the methods of Kumar et al.^24^ and Li et al.^25^, with modifications. Fresh leaves (0.2 g) were ground in 1 mL of 0.5 M trichloroacetic acid solution in an ice water bath, immersed in 5 mL of the solution, and shaken for 2 h at 0 °C. The solution was then centrifuged at 10,000 rpm for 10 min, after which 0.2 mL of the supernatant and 0.2 N H_2_SO_4_ (0.2 mL) were mixed together and subsequently boiled at 100 °C for 10 min. After cooling, 4 mL of anthrone reagent (0.2 g of anthrone in 100 mL of cold 95% sulfuric acid) was added to the above mixture, which was boiled at 100 °C for 10 min and then chilled again. The absorbance of the above solution was measured at 630 nm with a spectrophotometer, and the concentration was determined via a standard curve of trehalose.

#### Determination of antioxidant enzyme activities

Superoxide dismutase (SOD) activity was determined according to the methods of Beauchamp and Fridovich^26^, with one unit of activity as the amount of protein required to inhibit 50% of the initial reduction in nitro blue tetrazolium (NBT) under light. Peroxidase (POD) activity was assayed as described by Omran^27^. The activity of catalase (CAT) was assayed as described by Aebi^28^, and one unit was defined as the amount of enzyme required to decompose 1 μmol hydrogen peroxide min^−1^.

#### Gas exchange measurements

An LI-6400 (LI-COR Corporation of America) portable instrument was used to measure the gas exchange parameters, and the instrument automatically recorded the net photosynthesis rate (Pn), stomatal conductance (Gs), transpiration rate (Tr), intercellular carbon dioxide concentration (Ci) and stomatal limitation (Ls) value. The water-use efficiency (WUE) was calculated as Pn/Tr. The environmental light intensity during the tests was 1000 μmol m^−2^ s^−1^, the leaf temperature was 25 °C, the CO_2_ concentration was 400 μmol mol^−1^, and the relative humidity was 75%. The mature leaves from the middle of the plants were selected for testing, and five leaves were tested for each plant. The tested plants were pre-adapted to light, when the ‘PHOTO’ value tends to be stable (the fluctuation is generally less than 0.5 and lasts for more than 15 s), we save the measurement data.

#### Observations of leaf structure

The paraffin method was applied according to Li and Zhang^29^. On the 30th day of drought treatment, mature leaves of each treatment were randomly selected, and small pieces (1 cm × 3 mm) were cut from the part between the main vein to the margin of the leaf with a double-sided blade. The sample segments were then fixed in FAA stationary liquid (5% formalin, 5% acetic acid, and 90% ethanol) at 4 °C. The fixed segments were dehydrated in a graded series of ethanol solutions and further treated for transparency and for paraffining, embedding, production, dyeing and sealing. The thickness of each slice was 8 μm. The segments were then double stained with safranin O-fast green. The cell structure of the samples was observed and imaged with an optical microscope (DS-U3, Nikon, Japan), and the microstructure of the leaves was observed and imaged by a transmission electron microscope (H-600IV, Hitachi, Japan). The thickness of the leaves, epidermis, and cuticle was measured by microscope graticules, and the values were the average of 40 measurements.

### Statistical analyses

All the data were analyzed using SPSS 20.0 statistical software (IBM Corporation, Armonk, NY, USA). The data were analyzed by one-way analysis of variance together with the least significant difference (LSD) test at the 5% confidence level.

## Results

### Growth

Mild and moderate drought stress had no significant effect on plant height, which was 13.17% (*P* < 0.05) lower than that of the control only at 30 days of moderate stress. Severe drought stress significantly inhibited plant height, which was significantly lower than that of the control throughout the whole period, and the reduction increased with the increase in stress duration (Table 3). The lateral growth of plants was inhibited by drought stress, but there was no discernible pattern. At the initial stage of stress (10 d), the stem diameter under each treatment was significantly lower than that under the control treatment; on the 20th day, the stem diameter under T3 alone was significantly lower than that under the control; on the 30th day, the values under both T2 and T3 were significantly lower than the values under the control (Table 3). The effect of drought stress on root growth was different from that on shoot growth, which showed a certain promoting effect, but with the extension of stress duration, the promotion effect gradually diminished. In the early stage of stress (10 d), the root lengths under the T1 and T2 treatments were 38.92% (*P* < 0.05) and 42.89% (*P* < 0.05) higher than those under control, respectively; although the root length under the T3 treatment was lower than under T1 and T2, it was still significantly higher than that of the control. In the middle stage of stress (20 d), the root length under the T1 treatment was the largest, which was significantly higher than that under the other treatments, while the root length under the T3 treatment was not distinctly different from that of the control. In the late stage of stress (30 d), mild and moderate drought stress promoted root growth, but there was no significant difference from the control, while severe drought stress showed no significant inhibition of root growth (Table 3).

**Table 3:** Effects of drought stress on the plant height, root length and stem diameter of *Phedimus aizoon* L.

	Treatment	10 d	20 d	30 d
Plant height (cm)	Control	14.3 ± 0.4a	15.8 ± 1.3a	18.8 ± 0.9a
	T1	13.5 ± 0.7a	16.2 ± 0.5a	18.4 ± 0.5a
	T2	13.0 ± 1.1a	14.9 ± 0.3a	16.3 ± 0.3b
	T3	11.1 ± 0.7b	11.9 ± 0.7b	12.2 ± 0.6c
Stem diameter (cm)	Control	0.48 ± 0.01a	0.45 ± 0.05a	0.49 ± 0.03a
	T1	0.43 ± 0.01b	0.50 ± 0.01a	0.44 ± 0.03ab
	T2	0.42 ± 0.02b	0.49 ± 0.03a	0.41 ± 0.03b
	T3	0.42 ± 0.02b	0.39 ± 0.01b	0.39 ± 0.01b
Root length (cm)	Control	9.8 ± 0.8c	15.3 ± 0.8c	16.5 ± 1.4ab
	T1	13.7 ± 1.1a	18.1 ± 0.6a	17.7 ± 0.5a
	T2	14.1 ± 0.2a	16.7 ± 0.2b	17.0 ± 0.5a
	T3	12.2 ± 0.5b	15.1 ± 0.7c	15.3 ± 1.0b

The data are the means ± SDs of three replications. Use different letters to indicate significant differences in average values between the treatments within each individual time (*P* ≤ 0.05) based on Duncan’s multiple range test.

Control, 75–80% of the MWHC; T1, 55–60% of the MWHC; T2, 40–45% of the MWHC; T3, 20–25% of the MWHC.

### Relative water content

**Figure 1:**
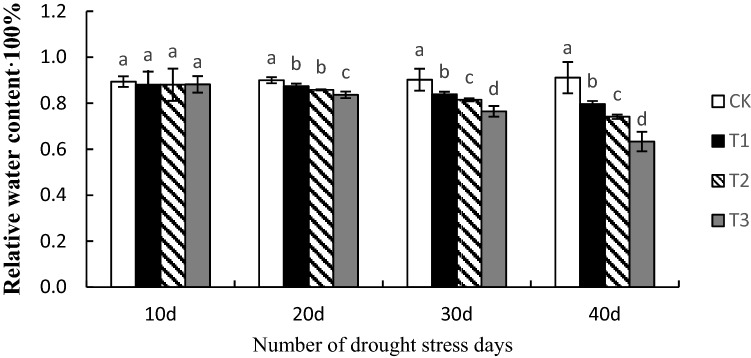
Effects of drought stress on the relative water content of *Phedimus aizoon* L. leaves. *Note*: The small letters mean significant differences at the *P* < 0.05 level.

The first 10 days of drought stress had no effect on the relative water content of the plants. The changes were detected on the 20th day, the relative water content of mild and moderate stress decreased, and the rate of decrease under severe stress was the greatest. By the 30th day of drought stress, there were significant differences between each treatment, and by the 40th day of drought stress, the difference was even more pronounced. Plant leaf water loss became increasingly severe with prolonged drought stress duration and increased stress intensity (Fig. 1).

### Leaf anatomical structure on the 30th day of drought stress

**Figure 2:**
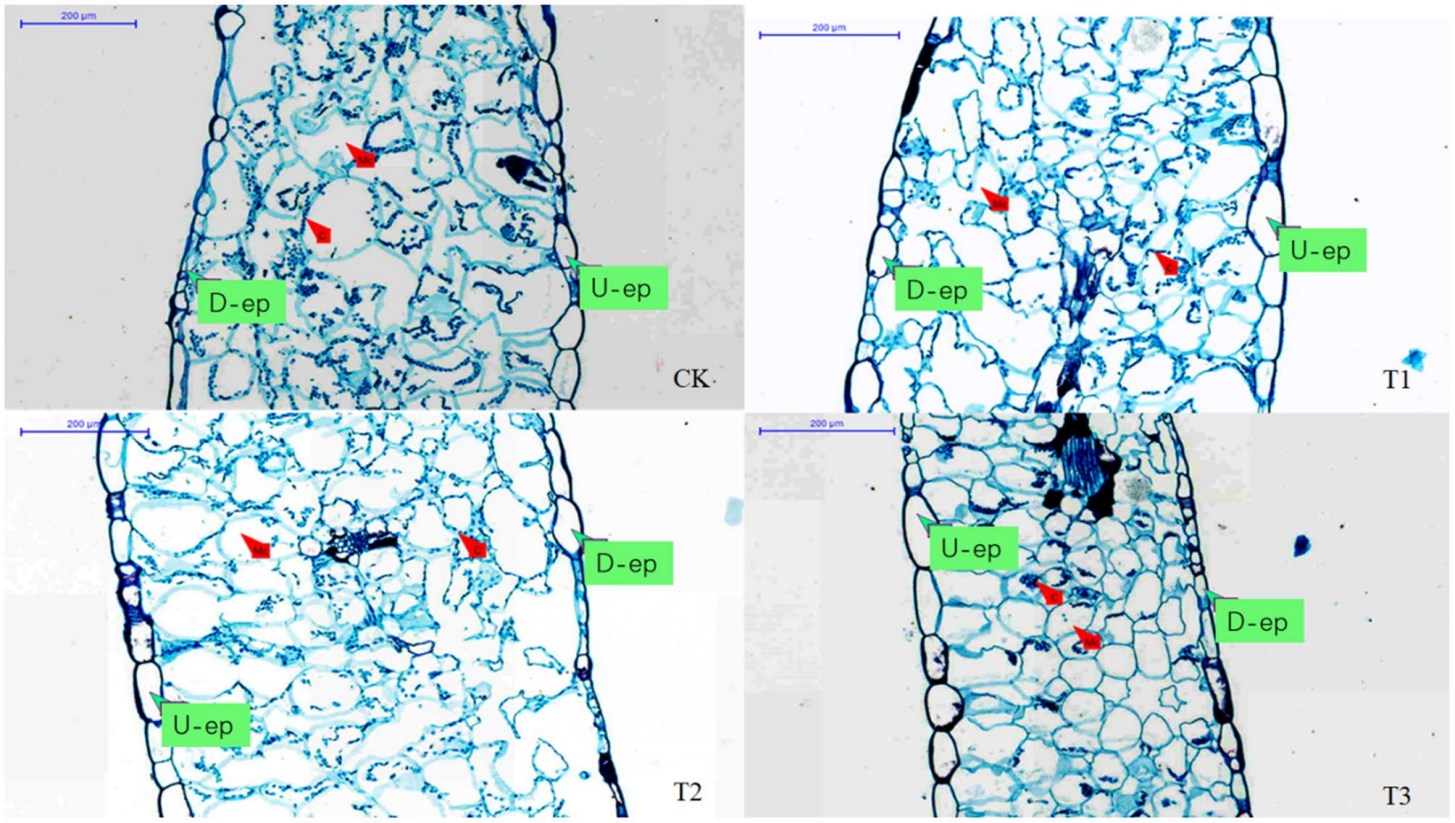
Changes in the anatomical structure of *Phedimus aizoon* L. leaves on the 30th day of drought stress. *Notes*: U-ep, upper epidermis; D-ep, lower epidermis; Mc, mesophyll cell; C, chloroplast.

The mesophyll cells of the leaves of the control plants were loose and well hydrated, and the individuals cells were large. Because of too much water in the cells, the effect of dehydration fixation was poor when the slices were made. However, as the intensity of drought stress increased, the arrangement of mesophyll cells gradually changed from loose to tight, and they gradually shrank and lost water. Especially under severe drought stress, the spaces between mesophyll cells decreased, the volume of cells decreased, leaf fixation after water loss improved, and the leaf slices were more complete. At the same time, the chloroplasts in the mesophyll cells were uniformly distributed along the cell wall under suitable moisture conditions but gradually gathered to the middle of the cells with the intensifying of drought stress. Under severe stress, the chloroplast envelope dissociated, the chloroplasts partially merged, and there was no complete chloroplasts in the cells (Fig. 2).

The results of leaf anatomical structure statistical analysis showed that, under mild drought stress, leaf thickness was 3.55% (*P* < 0.05) times higher than that of the control, while the leaf thickness decreased under moderate and severe drought stress and was significantly lower than that of the control, especially under severe stress (a decrease of 25.34% compared with that of the control). The upper epidermis thickness was significantly lower than that of the control only under severe drought stress. Under mild drought stress, there was no obvious change in the thickness of the lower epidermis, but under moderate and severe stress, it was 40.87% (*P* < 0.05) and 42.59% (*P* < 0.05) lower than that of the control, respectively. Second, the difference in cuticle thickness among the treatments was not significant, but there was a tendency for increased thickening with increasing stress (Table 4).

**Table 4:** Statistical analysis of the leaf anatomical structure of *Phedimus aizoon* L. under drought stress.

	Control	T1	T2	T3
Leaf thickness (μm)	740.56 ± 5.33b	766.81 ± 4.28a	718.59 ± 3.34c	552.84 ± 10.79d
Thickness of the upper epidermis (μm)	38.41 ± 7.25a	32.45 ± 3.18a	36.56 ± 1.99a	28.77 ± 2.26b
Thickness of the lower epidermis (μm)	24.56 ± 7.73a	23.15 ± 3.24a	14.52 ± 3.10b	14.10 ± 0.49b
Cuticle thickness (μm)	3.72 ± 0.51a	3.96 ± 0.69a	4.24 ± 0.87a	4.33 ± 0.74a

### Lipid peroxidation

**Figure 3:**
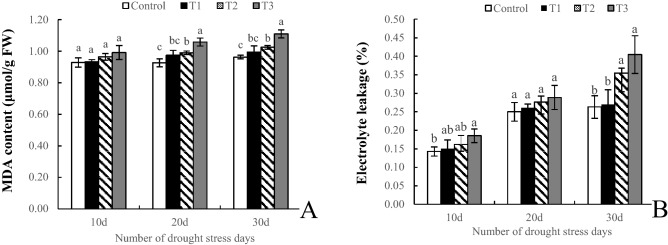
Effects of drought stress on membrane lipid peroxidation of *Phedimus aizoon* L. *Note*: MDA content (**A**), electrolyte leakage (**B**).

At the initial stage of stress (10 d), there was no significant difference in MDA content among any of the treatments. At 20 d, the con tents under the T2 and T3 treatments were significantly higher than those under the control. The change in MDA content at 30 d was the same as that at 20 d, and the MDA content in the T2 and T3 treatments increased by 6.43% (*P* < 0.05) and 15.24% (*P* < 0.05), respectively, compared with that in the control (Fig. 3A). After drought stress for 10 days, the electrolyte leakage rate was significantly higher only in the T3 treatment compared with the control. At 20 d, there was no significant difference among the treatments. At the later stage of stress (30 d), although there was no significant difference between T1 and the control, the leakage rate under both T2 and T3 were significantly higher than that under the control, with increases of 34.92% and 53.89%, respectively, compared with the control (Fig. 3B).

### Osmoregulatory substances

**Figure 4:**
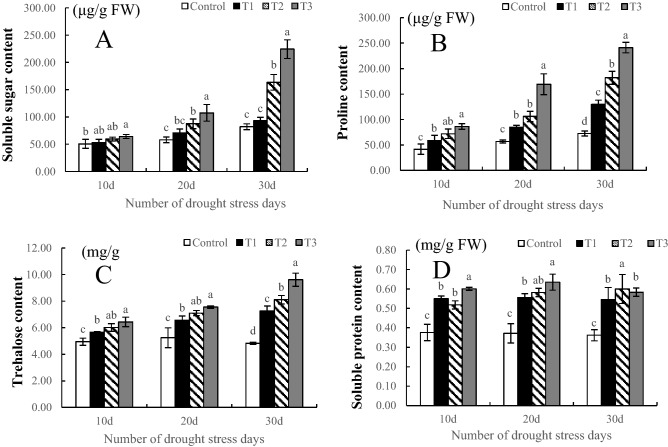
Effects of drought stress on the osmotica of *Phedimus aizoon* L. *Notes*: soluble protein content (**A**); proline content (**B**); trehalose content (**C**); soluble protein content (**D**).

The changes in soluble sugar content, proline content and trehalose content were essentially the same throughout the drought treatment and increased with increasing drought stress. The soluble sugar content under T1 showed no significant difference with that under the control during the whole stress period, and the contents under T2 and T3 were significantly higher than those under the control at both 20 d and 30 d (especially at 30 d), the values of which were 99.40% (*P* < 0.05) and 173.28% (*P* < 0.05) higher, respectively, than those under the control (Fig. 4A). The proline content in all treatments was significantly higher than that in the control throughout the period, and the higher the stress intensity was, the higher the proline content and the greater the increase. The content of proline in T3 was the highest at 10 d, 20 d and 30 d of stress, the contents of which were 107.52% (*P* < 0.05), 197.41% (*P* < 0.05) and 232.27% (*P* < 0.05) higher than that in the control, respectively (Fig. 4B). Consistent with the change in proline content, the trehalose content in all stages of the drought treatment was significantly higher than that in the control. At 30 d of drought stress, the difference among treatments was significant, especially in T3, where the trehalose content was the highest and was 199.37% (*P* < 0.05) higher than that in the control (Fig. 4C). The change in soluble protein content in the drought treatment was small in the different periods, and there was no obvious change trend; however, the soluble protein content was significantly higher than that in the control (Fig. 4D).

### Antioxidant enzyme activity

**Figure 5:**
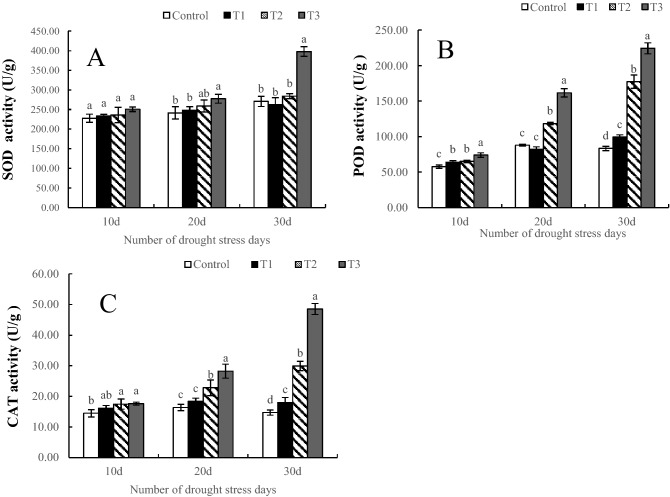
Effects of drought stress on the antioxidant enzyme activities in *Phedimus aizoon* L. *Notes*: SOD activity (**A**), POD activity (**B**), CAT activity (**C**).

With increasing drought intensity and time, the activity of antioxidant enzymes gradually increased. During the whole period of drought stress, the increase in SOD activity was small, except under the T3 treatment, and there was no significant difference in SOD activity between the other treatments (Fig. 5A). The changes in POD activity coincided with those of CAT activity under treatment for 10 days. Although there were significant differences between different treatments, the change range was small. At 20 days, although there was no significant difference in activity between T1 and the control, the differences among T2, T3 and the control were all significant. At 30 days of drought stress, the activities of POD and CAT were significantly different among all the treatments, and both SOD and CAT activity increased with increasing drought stress. In particular, the activity under the T3 treatment was significantly higher than that under the other treatments; the levels of SOD and CAT activity were respectively 169.81% and 230.47% higher than those of the control at 30 d (Fig. 5B,C).

### Photosynthesis parameters

**Figure 6:**
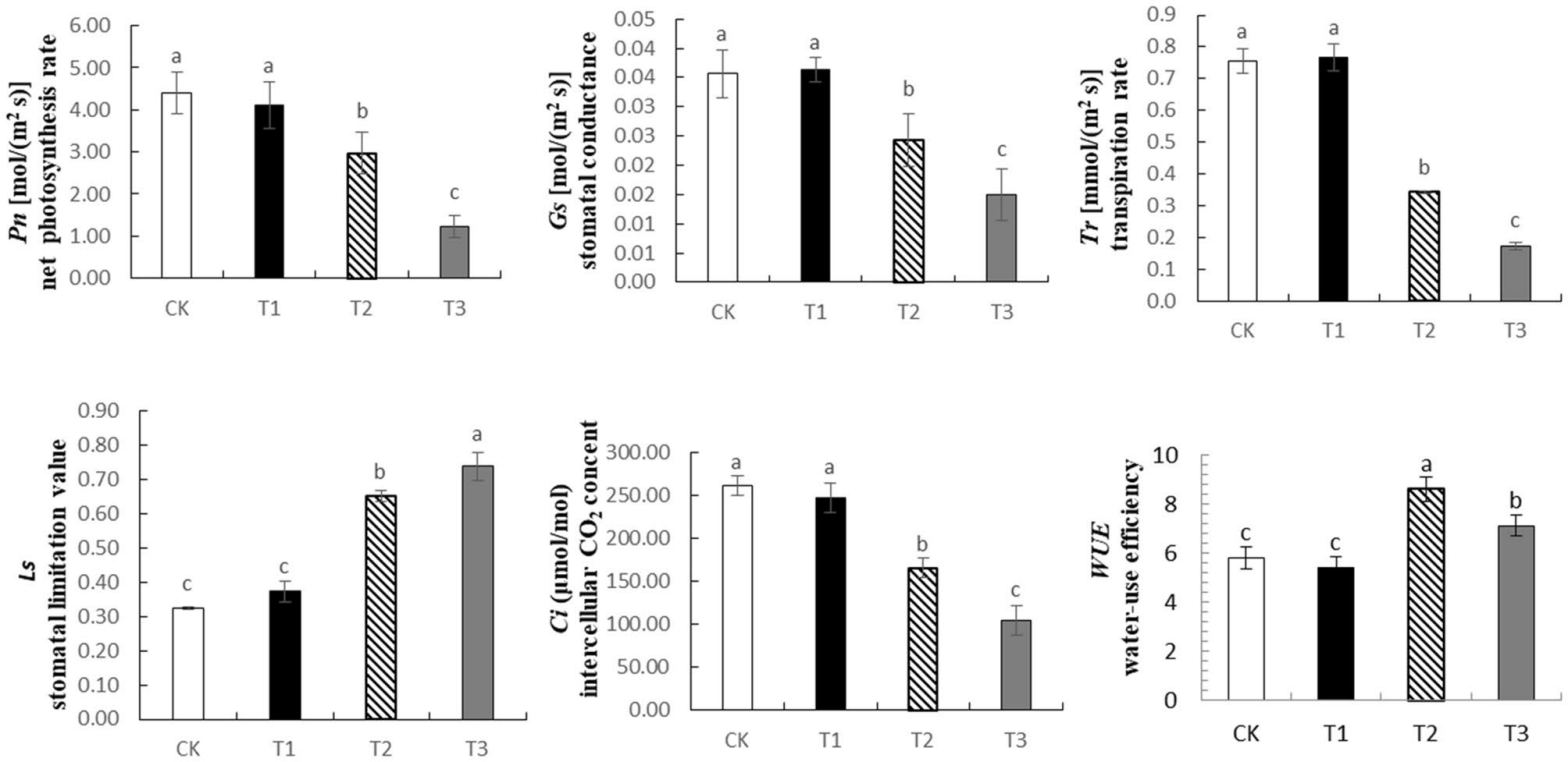
Effects of drought stress on photosynthesis parameters of *Phedimus aizoon* L. *Pn,* net photosynthesis rate; *Ls*, stomatal limitation value; *Tr*, transpiration rate; *WUE*, water-use efficiency; *Ci*, intercellular CO_2_ concentration; *Gs*, stomatal conductance.

On the 30th day of drought treatment, the photosynthesis parameters of *Phedimus aizoon* L. changed significantly. The net photosynthesis rate (*Pn*), stomatal conductance (*Gs*), transpiration rate (*Tr*), and intercellular carbon dioxide concentration (*Ci*) were negatively correlated with stress intensity, while the stomatal limitation (Ls) value was positively correlated with stress intensity. Especially under severe drought, the *Pn*, *Gs*, *Tr*, and *Ci* of plants decreased by 71.15%, 57.98%, 76.83% and 60.24%, respectively, while the Ls increased by 127.66%. Under moderate drought, the water-use efficiency (*WUE*) of the plants was significantly higher than that of the plants under the other treatments (Fig. 6).

### Stomatal aperture

**Figure 7:**
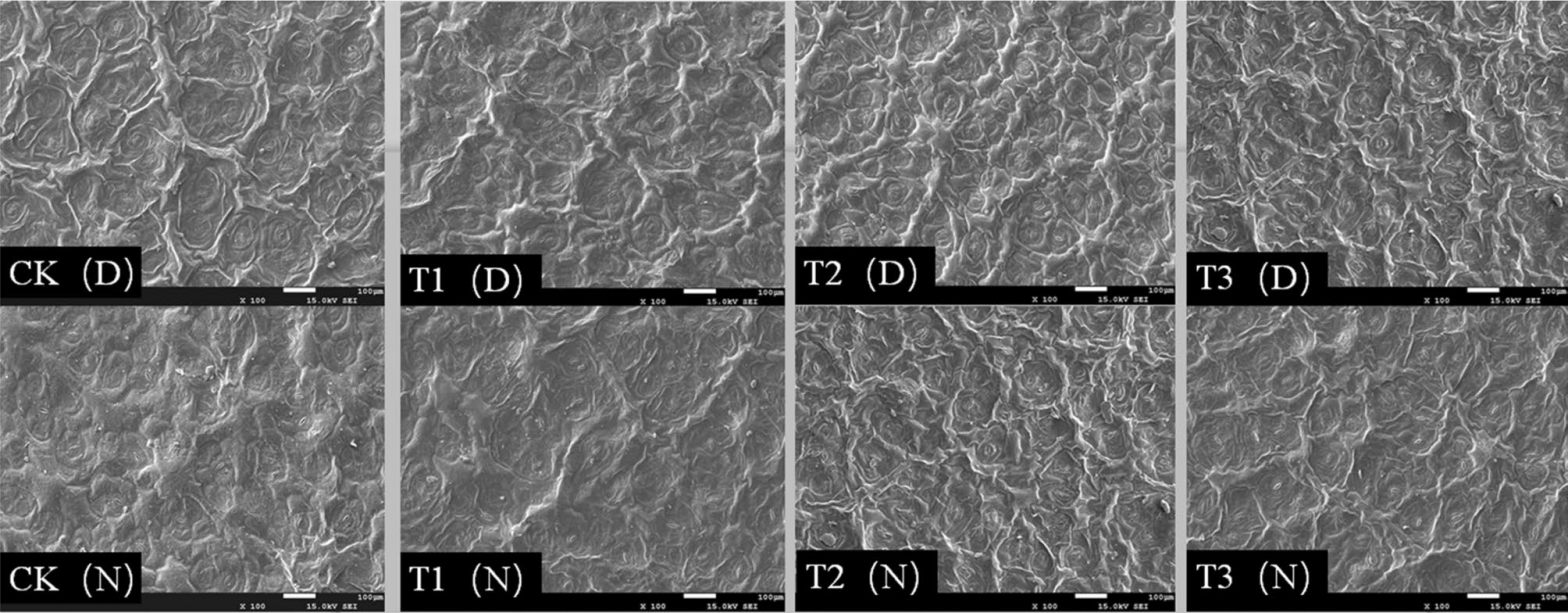
Scanning electron micrographs of *Phedimus aizoon* L. leaves under drought stress (× 100).

**Figure 8:**
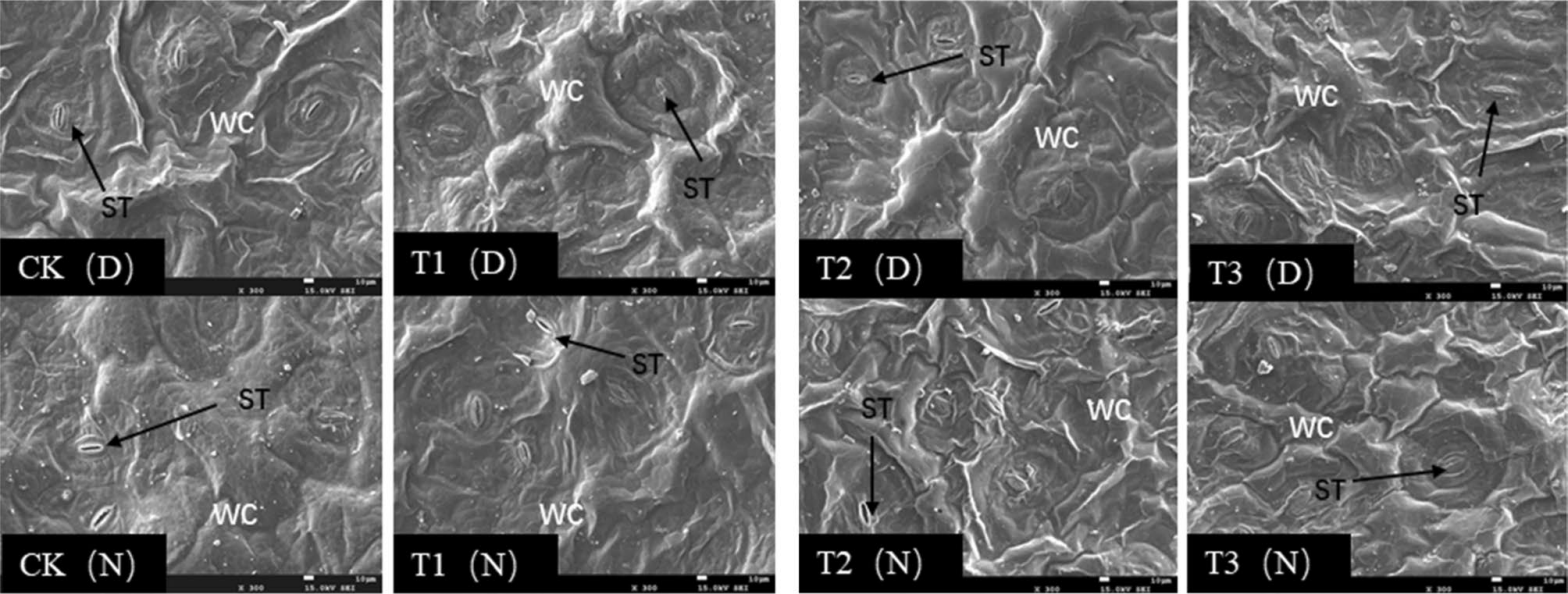
Scanning electron micrographs of *Phedimus aizoon* L. leaves under drought stress (× 300).

**Figure 9:**
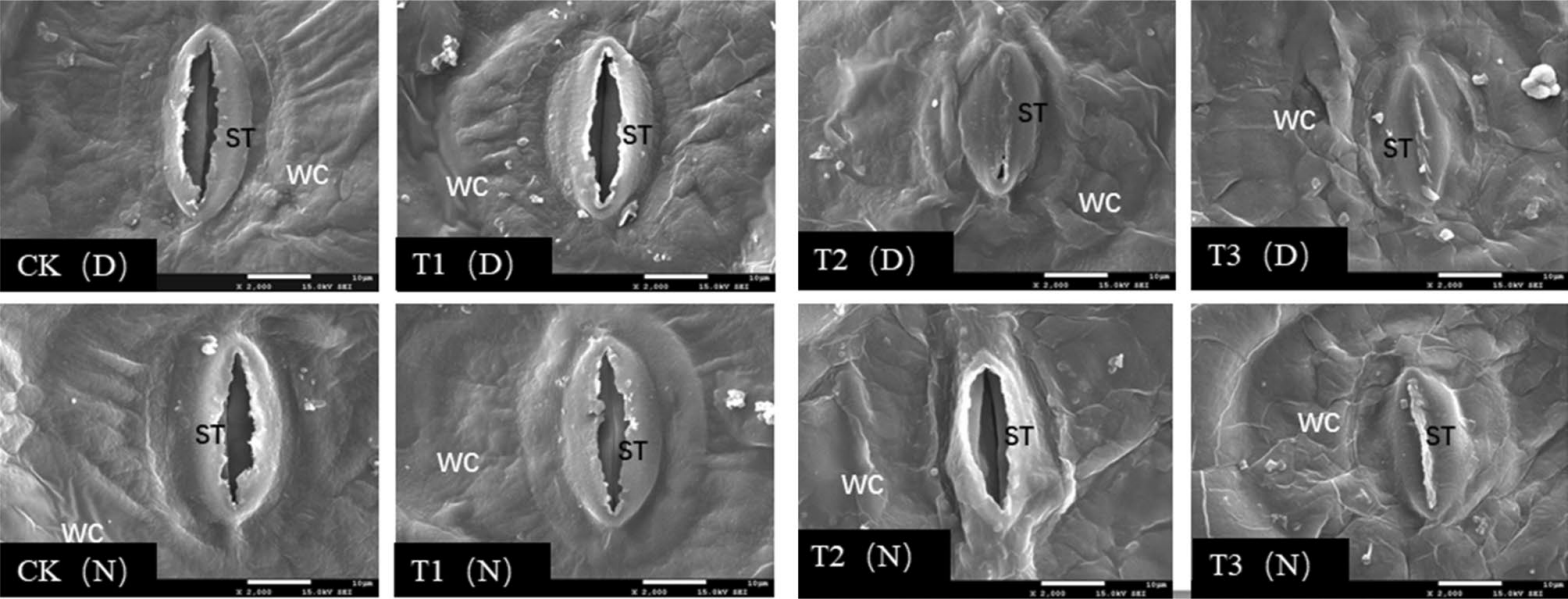
Scanning electron micrographs of *Phedimus aizoon* L. leaves under drought stress (× 2000). ST, stoma; WC, wax coat; D, daytime (11:00 a.m.); N, nighttime (11:00 a.m.).

We observed the stomata of *Phedimus aizoon* L. plants during the day and night (11:00 a.m. and 11:00 p.m.) at the 30th day of drought stress. As shown in Figs. 7 and 8, scanning electron microscopy revealed that in the CK and T1, the stomata were open during the day and night, and there was no significant difference in the surface wax layer. However, under the T2 treatment, the number of open stomata decreased during the day; the stomata were almost in a closed state, stomatal opening decreased, and the guard cells were slightly deformed and wrinkled. At night, the conditions were similar to those of the CK and T1; however, the waxy layer thickness increased, and the number of waxy crystals increased. Under the T3 treatment, we observed that the waxy layer on the leaf surface increased and thickened, the guard cells of stomata shrank, and the volume of stomata decreased (Fig. 9). The stomata were closed during the day and less open at night.

### Chloroplast and mitochondrial microstructure

**Figure 10:**
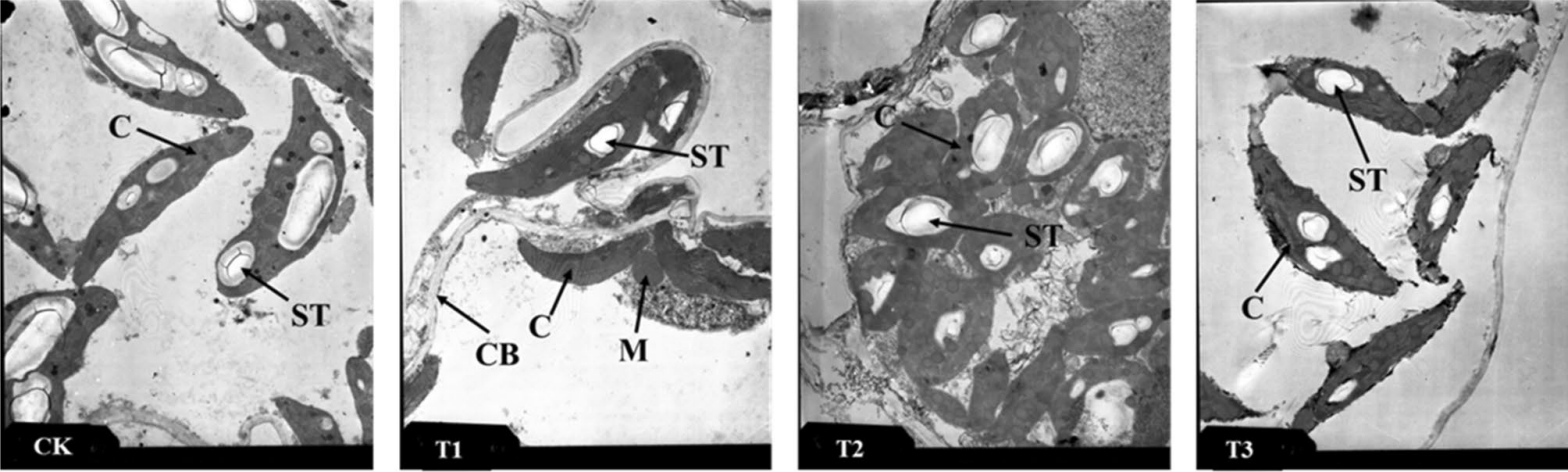
TEMs of *Phedimus aizoon* L. leaves under drought stress (× 5000). C, chloroplast; ST, starch grain; M, mitochondrion; CB, cytomembrane.

**Figure 11:**
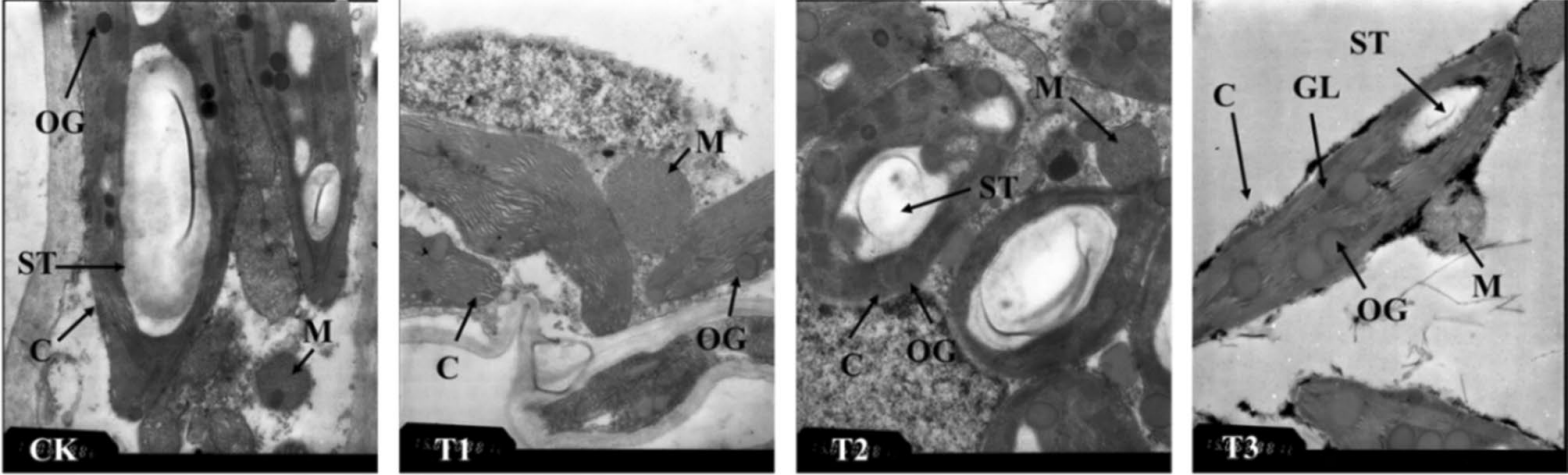
TEMs of *Phedimus aizoon* L. leaves under drought stress (× 15,000). C, chloroplast; ST, starch grain; M, mitochondrion; CB, cytomembrane; OG, osmiophilic globule; GL, granum lamella.

As shown in Figs. 10 and 11, the chloroplasts of the plants were evenly distributed along the cell membrane under the CK in a spindle shape, and the thylakoid membranes were neatly and closely arranged, with starch grains embedded in them. The mitochondria were spherical and round and presented clear boundaries. With the intensifying of stress, the chloroplasts gradually shriveled, the boundaries were rough, the thylakoid membranes were loose, the lamellae were disrupted, the number of starch grains decreased, and the chloroplast volume decreased. The internal compartments of the mitochondria were dissolved, and they were swollen and white in color; the number of osmiophilic granules gradually increased. These findings indicate that the membrane system was irreversibly damaged by drought.

## Discussion

Growth is a comprehensive response of plants to drought stress and a reliable criterion for evaluating the degree of drought stress and the drought stress resistance of plants. Decreases in plant height and stem diameter are common phenomena under drought stress^30^. The results of this study showed that, under drought stress, the shoot growth of *Phedimus aizoon* L. was inhibited, and the stronger the degree of stress was and the longer the duration, the more significantly inhibited the plants were (Table 3). The main reason was that the lack of water led to clogging of vascular tissue and reduced cell elongation^31^. Roots are the main organs of plants for water absorption and are closely related to drought stress resistance. Root systems can adapt to drought stress by regulating their own growth and development, water absorption and transport. The root length proliferation of *Phedimus aizoon* L. was promoted under mild and moderate drought stress (Table 3), which was beneficial for the root system to absorb the deep water of the soil and improve the utilization rate, thus improving the drought tolerance of the plant^9^.

Throughout evolution, leaves have been sensitive to drought. Because of their plasticity, changes in leaf morphological structure will inevitably lead to changes in the physiological and biochemical characteristics of plants. Therefore, changes in leaf morphological traits can reflect the adaptability of plants to drought stress^32^. The thicker the plant leaves are, the greater the compactness of the tissue and the greater the water storage capacity and drought tolerance of the leaves^33^. *Phedimus aizoon* L. is a typical Phedimus plant species with strong drought tolerance, and its leaves are especially fleshy, which is closely related to the drought tolerance of the plants^34^. Through observations of leaf anatomical structure, it was found that, in the absence of drought stress, the leaves of *Phedimus aizoon* L. were thicker, and the mesophyll cells were large and irregular, arranged loosely, with large intercellular spaces and with a strong water storage capacity. Under moderate and severe stress, the mesophyll cells gradually aggregated and decreased in size, the chloroplasts gradually aggregated to the middle of the cells, and the upper epidermis became thinner (Fig. 2, Table 4). The purpose of these changes is to reduce the direct contact of plants with radiation, and reduce transpiration to preserve the limited water and make full use of it^35^. Especially under severe stress, the leaf thickness and upper epidermis thickness were significantly lower than those of the control, and the tissue was loosely to tightly arranged. As the drought intensified, water between the mesophyll cells was consumed; thus, the intercellular space shrank, the cells were tightly arranged after becoming compacted, and the cuticle gradually thickened (Fig. 2, Table 4), which indicated that the leaf tissue of *Phedimus aizoon* L. was strongly resistant to drought stress.

The plasma membrane is the initial location where plants are first damaged under stress. Under drought stress, the plasma membrane is damaged, which is characterized by increased plasma membrane permeability and partial electrolyte leakage^36^. At the same time, a large amount of reactive oxide species are produced in plants, which induce membrane lipid peroxidation and produce malondialdehyde, thus causing damage to plant cell membrane systems^37^. The MDA content and changes in plasma membrane permeability are important indicators reflecting the degree of plasma membrane damage. This study showed that, the MDA content increased with the duration of stress, but there was no difference between moderate and mild stress, although the electrolyte leakage increased with increasing duration and degree of drought stress, throughout the growth phase, the electrolyte leakage fluctuated significantly only at 30 d, and there were no significant difference between mild drought stress and the control from the beginning to the end (Fig. 3), indicating that *Phedimus aizoon* L. could withstand a certain degree and duration of water deficit.

The accumulation of osmotic regulatory substances (proline, soluble sugars and soluble protein) is one of the basic characteristics of plants to adapt to drought stress. Under drought stress, plants actively accumulate osmotic regulatory substances to increase the concentration of cell fluid, the main function of which is to maintain cell turgor, balance the infiltration of protoplasm and the external environment, and enable various physiological processes of cells to proceed normally. Moreover, it is generally believed that the drought stress resistance of plants is positively correlated with osmoticum content^38^. The contents of soluble sugars, proline and trehalose in the leaves of *Phedimus aizoon* L. significantly increased under drought stress, especially at 30 days of stress (Fig. 4A,B,C). The results of this study are similar to those of the study of Guo^39^, which also showed that under drought conditions, as the stress duration increased, the content of osmotic regulatory substances such as proline and soluble sugars increased sharply. The increase in these osmotic regulatory substances reduced the cell osmotic potential and ensured that *Phedimus aizoon* L. could continue to absorb water from the soil. However, the change in soluble protein content was slightly different from that of other osmotic regulatory substances. A possible reason is that, as the drought duration increases, the degree of stress intensifies, and the metabolism of plants is inhibited, resulting in the inability of soluble proteins to continue to increase and even decrease.

Oxidative stress usually accompanies drought stress, and the antioxidant defense system is one of the mechanisms in response to drought; this mechanism allows aerobic metabolism for energy for plant growth and development. The generation and clearance of intracellular reactive oxygen species (ROS) are normally in a state of dynamic equilibrium, but when plants are subjected to drought stress, this dynamic balance is disrupted^11^. Antioxidant enzymes such as superoxide dismutase (SOD), peroxidase (POD) and catalase (CAT) are effective at scavenging reactive oxygen species, preventing excessive ROS accumulation, and protecting plants from harm^40^. This study showed that, to remove excess ROS and reduce oxidative damage, *Phedimus aizoon* L. could maintain high antioxidant enzyme activity under different degrees of drought stress. Especially under severe drought stress and in the later period of stress, the activities of SOD, POD and CAT increased sharply (Fig. 5). The main reason is that the production of ROS in the plants increased sharply under these conditions; although this caused severe damage to the normal metabolism of *Phedimus aizoon* L., the plants could still remove these ROS by increasing their activity of antioxidant enzymes accordingly, showing their strong resistance to drought stress.

The biological activities of plants depend on stomata for gas exchange, and stomata are also channels for transpiration. The size, degree of opening and stomatal density directly affect the transpiration rate of plants^41^. Plants with facultative crassulacean acid metabolism (CAM) maximize performance by utilizing C3 or C4 photosynthesis under ideal conditions while temporarily switching to CAM under drought stress^42^. In this experiment, on the 30th day of moderate stress (Figs. 7, 8, 9), leaf stomata closed during the day and opened at night. The opening and closing state of the stomata determines the absorption of CO_2_. At this time, the intercellular CO_2_ concentration (Ci) significantly decreased (Fig. 6). The stomatal conductance (*Gs*) and transpiration rate (*Tr*) of the leaves significantly decreased, and the stomatal limitation (*Ls*) value significantly increased, leading to a decline in photosynthesis. Apparently, the metabolic pattern of the plant shifts to that of CAM cycling, which results in the absorption of CO_2_ and a reduction in evaporation. On the 30th day of severe stress, the stomata closed 24 h a day and were unable to absorb CO_2_, and the metabolic mode at that time was CAM idling, which occurs only under extreme drought stress; although the stomata were closed all day, the tissue acidity still fluctuates^43^. CAM idling is a kind of metabolic readiness, allowing rapid recovery from extreme droughts. This ability to recover quickly is particularly important, especially given that the plants in this experiment were grown in soils that were rapidly wetted and rapidly dried after being replenished with water^44^. For every gram of carbohydrate produced via CAM, Crassulaceae plants consume one-tenth as much water as C3 plants do. The CAM pathway not only reduces water loss through stomata but also promotes water absorption by plants^45^. This coincides with the improvement of WUE (water-use efficiency) on the 30th day of moderate drought in this experiment. The results of ^14^CO_2_ pulse-chase experiments suggest that, in well-watered plants, a CAM pattern of carbon flow already exists; hence, water stress might enhance latent CAM rather than induce it^46^. Although this was not studied in this experiment, we agree with this view because stomata also opened widely at night in the control group and under mild stress on the 30th day of drought. Under drought stress, *Phedimus aizoon* L. can reduce water loss by regulating its stomatal state and enhance latent CAM, which is another important reason for the improvement of drought stress resistance.

Chloroplasts and mitochondria are the two most important organelles for photosynthesis and respiration. Drought stress can alter the microstructure of plants. In this study, chloroplasts gradually converged to the middle of cells on the 30th day of moderate and severe stress, and the chloroplasts shrank under severe stress, resulting in fragmentation of the granum lamella, internal dissolution, and disruption of membrane structure, all of which are consistent with the increase in MDA content and electrolyte leakage of the leaves. In Katya Georgieva’s study^47^, the authors found an inverse staining pattern that had not previously been reported. This indicates that the thylakoid lumen is filled with an electron-dense substance (dense luminal substance (DLS)). This is possibly a phenolic substance that provides protection against oxidative damage, and a dense phenolic substance was thought to prevent conformational changes of membrane lipids^48, 49^. In this experiment, although the transmission electron microscope images were not able to clearly observe DLSs (Figs. 10, 11), changes in staining could be generally observed. The light staining of DLSs indicates that the membrane is under drought stress damage and is protected by DLSs. At the same time, an increase in osmiophilic granules and the fragmentation and dissolution of starch granules were clearly observed. These substances increased the osmotic potential of cells, which contributed to water absorption.

## Conclusions

**Figure 12:**
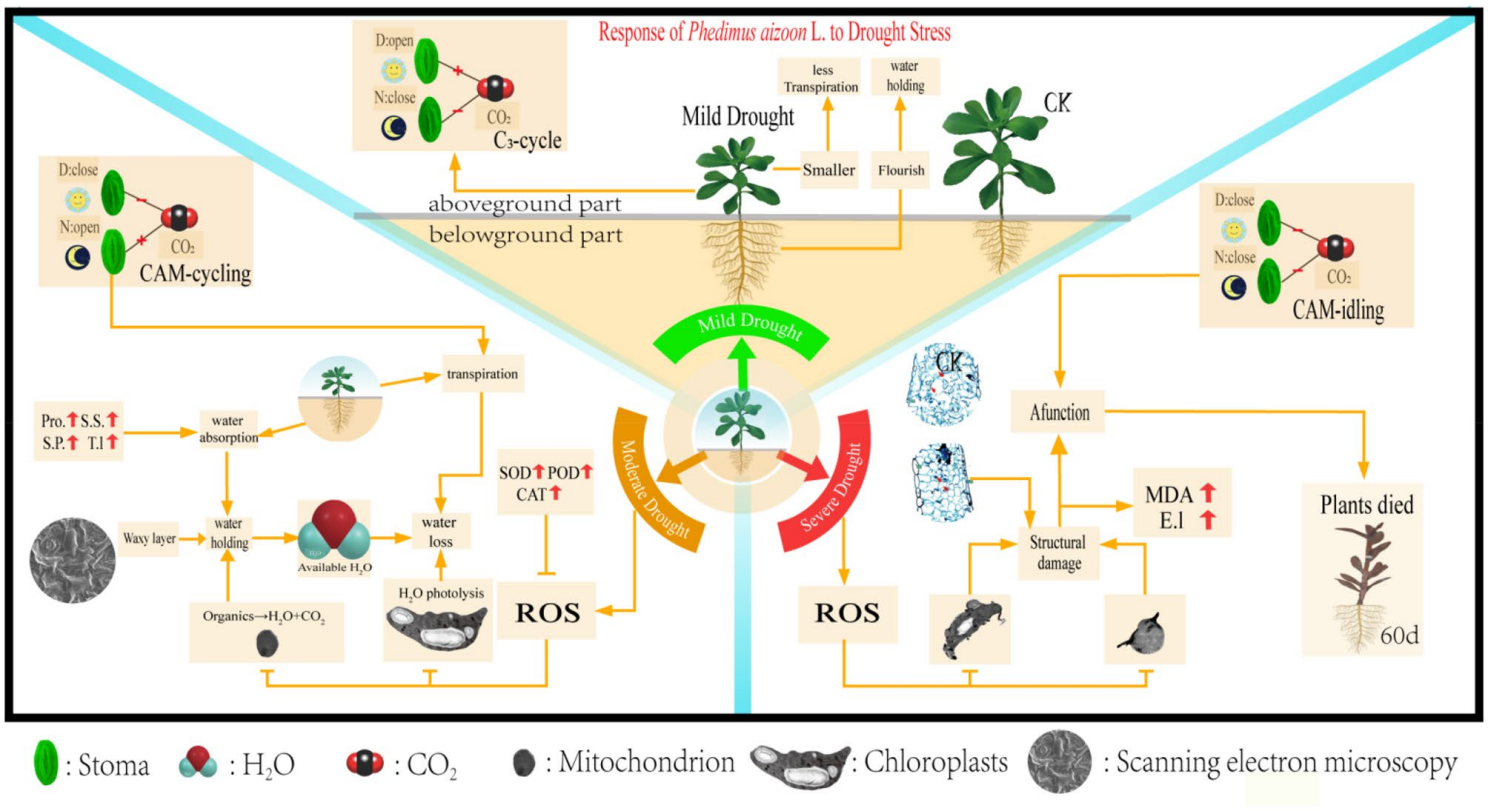
Response of *Phedimus aizoon* L. to drought stress. D, day; N, night; Pro, proline; S.S., soluble sugars; S.P., soluble protein; T.l., trehalose; MDA, malonaldehyde; E.l., electrolyte leakage; ROS, reactive oxygen species.

In summary (Fig. 12), when *Phedimus aizoon* L. was subjected to drought stress, the structure and physiology of the plants changed accordingly to adapt to drought. Mild drought stress had little effect on *Phedimus aizoon* L. However, under moderate and severe stress, plant growth was significantly inhibited; at the same time, the plants also displayed corresponding responses to drought. The increase in osmotic regulatory substances increased the water absorption capacity of the plants, while the increased activity of antioxidant enzymes alleviated the damage caused by ROS generated in response to drought stress. The thickening of the wax layer and the closing of stomata increased the water retention capacity of the plants, and CAM cycling reduced water loss through the stomata and promoted water absorption by the plants. CAM enables plants to rapidly recover from extreme drought. All of these positive changes constitute the drought stress resistance mechanism of *Phedimus aizoon* L. Although *Phedimus aizoon* L. has strong drought stress resistance, when these plants are cultivated, the soil available water content (W_hc_ − W_pwp_) should not be less than 27%.

## Supplementary information

Supplementary Information.
